# Effect of intestinal tapeworms on the gut microbiota of the common carp, *Cyprinus carpio*

**DOI:** 10.1186/s13071-019-3510-z

**Published:** 2019-05-22

**Authors:** Pei P. Fu, Fan Xiong, Wen W. Feng, Hong Zou, Shan G. Wu, Ming Li, Gui T. Wang, Wen X. Li

**Affiliations:** 1 Key Laboratory of Aquaculture Disease Control, Ministry of Agriculture, and State Key Laboratory of Freshwater Ecology and Biotechnology, Institute of Hydrobiology, Chinese Academy of Sciences, Wuhan, 430072 People’s Republic of China; 2 University of Chinese Academy of Sciences, Beijing, 100049 People’s Republic of China

**Keywords:** *Khawia japonensis*, *Atractolytocestus tenuicollis*, Intestinal microbiota, *Cyprinus carpio*

## Abstract

**Background:**

Parasitic protozoans, helminths, alter the gut microbiota in mammals, yet little is known about the influence of intestinal cestodes on gut microbiota in fish. In the present study, the composition and diversity of the hindgut microbiota were determined in the intestine of common carp (*Cyprinus carpio*) infected with two tapeworm species, *Khawia japonensis* and *Atractolytocestus tenuicollis*.

**Results:**

The intestine contained a core microbiota composed of Proteobacteria, Fusobacteria and Tenericutes. Infection with the two cestode species had no significant effect on the microbial diversity and richness, but it altered the microbial composition at the genus level. PCoA analysis indicated that microbial communities in the infected and uninfected common carp could not be distinguished from each other. However, a Mantel test indicated that the abundance of *K. japonensis* was significantly correlated with the microbial composition (*P* = 0.015), while the abundance of *A. tenuicollis* was not (*P* = 0.954). According to Pearsonʼs correlation analysis, the abundance of *K. japonensis* exhibited an extremely significant (*P *< 0.001) positive correlation with the following gut microbiota taxa: *Epulopiscium*, *U114*, *Bacteroides*, *Clostridium* and *Peptostreptococcaceae* (0.8< *r *< 0.9); and a significant (*P *< 0.05) correlation with *Enterobacteriaceae*, *Micrococcaceae*, *Rummeliibacillus*, *Lysinibacillus* *boronitolerans*, *Veillonellaceae*, *Oxalobacteraceae*, *Aeromonadaceae* (negative), *Marinibacillus* and *Chitinilyticum* (0.4< *r *< 0.7).

**Conclusions:**

These results suggest that the composition of gut microbiota was somewhat affected by the *K. japonensis* infection. Additionally, increased ratios of pathogenic bacteria (*Lawsonia* and *Plesiomonas*) were also associated with the *K. japonensis* infection, which may therefore increase the likelihood of disease.

**Electronic supplementary material:**

The online version of this article (10.1186/s13071-019-3510-z) contains supplementary material, which is available to authorized users.

## Background

The gastrointestinal (GI) tract of vertebrates is inhabited by a vast array of organisms, including bacteria, protozoan and helminth parasites [1]. Gut microbiota co-evolve with their hosts and have important functional roles in metabolism, nutrition and immunity [2]. Sharing the same niche with the intestinal microbiota [2], the enteric helminths inevitably interact with the gut microbiota [1], but interactions between gut microbiota and parasites are usually overlooked.

Increasing evidence indicates that the presence of parasitic helminths (including nematodes, cestodes and digeneans) alters the composition and diversity of GI microbiota in mammals [1, 3, 4]. In laboratory experiments, infection with nematodes or cestodes can induce changes in the microbial composition in the intestines of humans and other mammals, sometimes even resulting in a reduced bacterial diversity [5–8]. However, more often it appears to have a negligible effect on the bacterial diversity [9–17]. In field experiments, however, natural infection by nematodes hardly produces any effects on the microbial diversity in mammals [18–20], except for one study, which found high microbial diversity in the presence of multiple helminths in wild rodents [21]. In summary, regardless of whether the diversity of microbiota is altered, its composition can be changed by experimental and natural helminth infection both in domestic and wild mammals.

In contrast to mammalian systems, we know very little about interactions between the GI microbiota and helminths in cold-blooded animals such as fish. Although the parasite load of the myxozoan *Tetracapsuloides bryosalmonae* in the kidney of salmonid fish exhibits a significant positive relationship with the richness of the GI microbiome [22], little else is known about interactions between microbiota and helminths in the digestive tract of fish.

The common carp, *Cyprinus carpio*, is widely distributed in rivers, lakes, reservoirs and ponds in East Asia (as well as North America, Europe and Africa). The tapeworms *Khawia sinensis*, *K. japonensis* and *Atractolytocestus* sp. are commonly found in the intestine of the common carp [23–26]. Tapeworms can rob the host of nutrients through the tegument and thereby restrict the host’s growth. However, neither pathological changes nor poor physiological state can be observed in common carp, even when heavily infected by *Khawia* sp. [24, 27]. It has been hypothesized that GI microbiota associated with the presence of tapeworms may help maintain the homeostasis of the digestive system [28]. Therefore, in this study, we aim to investigate whether the presence of tapeworms influences the composition and diversity of microbiota in the intestine of wild common carp.

## Methods

### Collection of fish and tapeworms

Live common carp (*n* = 23) were collected in April 2017 from Liangzi lake (30° 04′–30° 20′ N, 114° 31′–114° 42′ E), Hubei Province, China. Their intestinal tracts were aseptically removed from the abdominal cavity, and then divided into three fragments (foregut, midgut and hindgut). The foregut and midgut were used for the collection of tapeworms, and the hindgut was used for microbiota analysis.

The collected tapeworms were gently rinsed in saline, prefixed in 70% hot alcohol, and then taxonomically identified according to the morphological features of the scolex under a stereomicroscope [26]. Owing to the similar scolex morphology of *Atractolytocestus* spp., *A. tenuicollis* was identified using the number of testes [23]. Abundance and maturity of tapeworms were also recorded.

### Sample preparation and total bacterial DNA extraction

The intestinal content of each fish was gently squeezed into a sterile tube and thoroughly mixed. The samples were frozen immediately in a freezer and stored at − 80 °C. The total bacterial DNA was extracted from 200 mg of the hindgut content using QIAamp® DNA stool mini kit (Qiagen, New York, USA) according to the manufacturer’s instructions. The purity and concentration of genomic DNA were determined with a spectrophotometer (Nanodrop 8000; Thermo Fisher Scientific, Wilmington, USA). DNA was stored at − 20 °C for later use.

## *16S* rDNA amplification and Illumina high-throughput sequencing

The universal primer pair 515F (5′-GTG YCA GCM GCC GCG GTA-3′), with a unique 12-nt barcode at the 5′-end, and 909R (5′-CCC GYC AAT TCM TTT RAG T-3′) [29], were used to amplify the V4-V5 hypervariable region of the bacterial *16S* rDNA gene. The PCR reaction system (25 μl) contained 50 ng of DNA template, 1 μM of each primer, and 12.5 μl of 2× Go Taq Green Master Mix polymerase (Promega, Madison, USA). The PCR procedure was as follows: 5 min at 94 °C as an initial step, followed by 23 cycles of 30 s at 94 °C, 30 s at 55 °C and 90 s at 72 °C, with a final step of 10 min at 72 °C. Replicated PCR products of the same sample were assembled into a PCR tube and subjected to electrophoresis using a 2% agarose gel. The correct band (about 400 bp) was recovered by AidQuick Gel Extraction Kit (Aidlad Biotech, Beijing, China). A spectrophotometer (Nanodrop 8000) was used to determine the DNA concentration and purity. All samples were pooled, with an equal molar amount from each sample, and sent to Novogene Bioinformatics Technology Co. for PCR-free library construction. Sequencing was performed using the PE250 strategy on an Illumina Hiseq 2500 platform. The sequences are available in the NCBI SRA database under the accession number SRP158810.

### Sequence data processing

The raw sequenced data were processed using QIIME Pipeline-Version 1.8.0 (http://qiime.org/) [30]. Overlapping paired-end reads were merged using FLASH-1.2.8 software [31]. Only the merged sequences with high-quality reads (length > 250 bp, without ambiguous bases BN, and average base quality score > 30) were used for further analysis. All sequences were trimmed and assigned to each sample on the basis of their barcodes (barcode mismatches = 0). Chimeras were removed using the UCHIME algorithm [32]. Non-chimera sequences were subsampled to the same sequence depth (14,581 reads per sample) using daisychopper.pl [33]. This subset of sequences was clustered into OTUs at a 97% identity threshold using CD-HIT [34]. Singletons were filtered out. Operational taxonomic unit (OTU) identities were assigned in Greengenes database (release 13.8) [35] using UCLUST [36]. Sequences classified as unassigned and C_Chloroplast were removed.

The following alpha diversity indices were calculated: Chao1, ACE, Simpson and Shannon index. Linear discriminant analysis coupled with effect size (Lefse) (http://huttenhower.sph.harvard.edu/galaxy) was used to study the significance of species differences at the genus level [37]. Principal coordinates analysis plots (PCoA) were used to visualize similarities between groups with weighted_unifrac distance [38]. Permutational multivariate analyses of variance (PERMANOVA) were performed to test the significance of differences between groups applying the Vegan package in R. The Pearsonʼs correlation coefficient was used to investigate the degree of linear correlation between the abundance of tapeworms and the abundance of bacteria using PAST 2.16 [39]. Association between the abundance of tapeworms and intestinal microbial community was analysed by a Mantel test using PASSaGE 2 [40]. A Venn diagram of shared and unique OTUs was used to describe the similarities and differences between infected and uninfected common carp (http://bioinfogp.cnb.csic.es/tools/venny/index.html).

### Statistical analyses

Statistical analyses were performed using SPSS20 (IBM Corporation, Armonk, NY, USA). Pairwise comparisons between infected and uninfected groups were assessed using Student’s t-test at the 0.05 significance threshold.

## Results

### Tapeworm infection prevalence and mean intensity

Two species of the Caryophyllidea, *Khawia japonensis* and *Atractolytocestus tenuicollis* (Additional file 1: Figure S1; Additional file 2: Figure S2), were found in the intestine of common carp (Additional file 3: Table S1). The prevalence and mean abundance (± standard deviation, SD) were respectively 60.9% (14/23) and 3.9 ± 6.9 (0–31) for *K. japonensis*, and 73.9% (17/23) and 7.3 ± 9.5 (0–31) for *A. tenuicollis* (Table 1).

**Table 1:** Abundance of *Atractolytocestus tenuicollis* and *Khawia japonensis* in the intestine of the common carp (*Cyprinus carpio*)

Sample ID	*A. tenuicollis*	*K. japonensis*	Group 1	Total number	Group 2
F17	7	12	KJ(+)	19	Infected
F18	1	1		2	
F21	0	2		2	
F22	8	31		39	
F23	1	1		2	
F24	4	3		7	
F26	1	9		10	
F27	1	1		2	
F28	3	8		11	
F30	21	1		22	
F34	0	1		1	
F36	14	8		22	
F37	25	5		30	
F38	2	6		8	
F19	18	0	KJ(−)	18	
F20	3	0		3	
F32	22	0		22	
F35	6	0		6	
F39	31	0		31	
F25	0	0		0	Uninfected
F29	0	0		0	
F41	0	0		0	
F42	0	0		0	

*Key*: KJ(+), infected by *K. japonensis*; KJ(−), uninfected by *K. japonensis*; Infected, infected by both tapeworms; Uninfected, infected by neither of the two tapeworms

### Microbial composition in infected and uninfected common carp

**Fig. 1:**
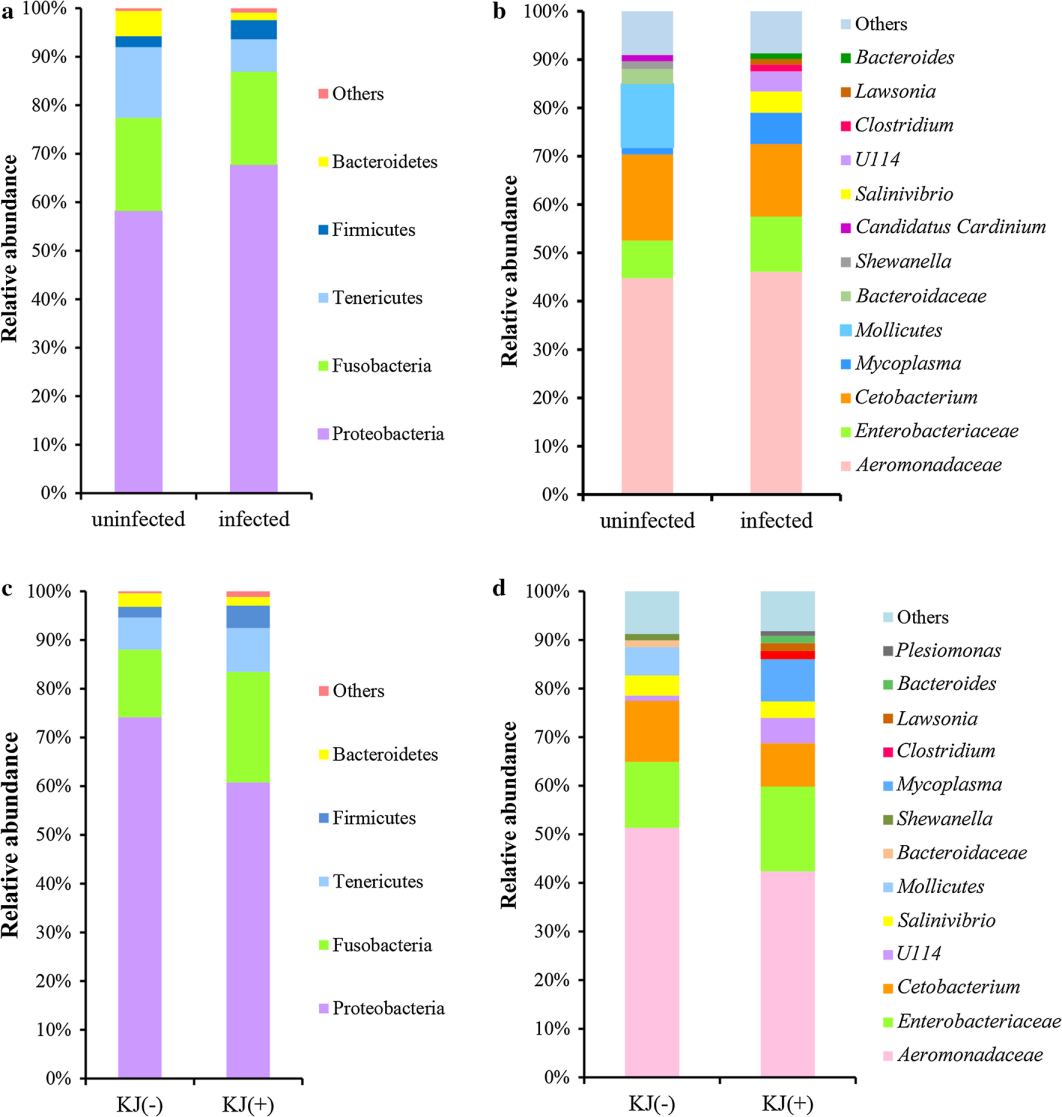
Microbial composition in the intestine of common carp. **a** the dominant phyla in uninfected and infected groups. **b** the dominant genera/families in uninfected and infected groups. **c** the dominant phyla in KJ(−) and KJ(+) groups. **d** the dominant genera/families in KJ(−) and KJ(+) groups. “Others” includes the sum of different taxa with an abundance less than 1% in the samples. *Abbreviations*: KJ(+), infected by *Khawia japonensis*; KJ(−), uninfected by *K. japonensis*

**Fig. 2:**
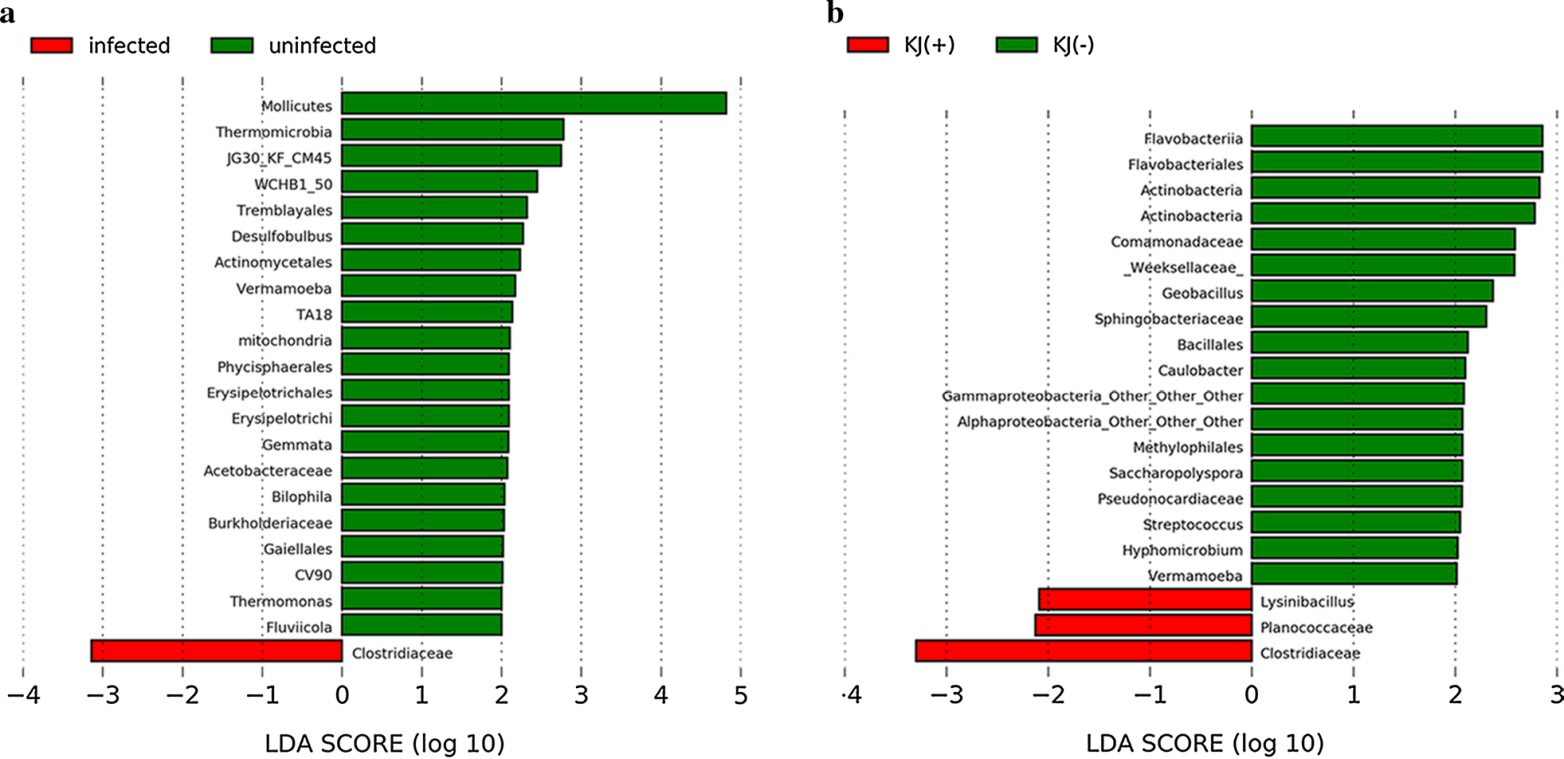
Bacterial taxa with significant differences (LDA score > 2.0) in the relative abundance identified by Lefse in uninfected and infected groups (**a**), and KJ(−) and KJ(+) groups (**b**)

**Fig. 3:**
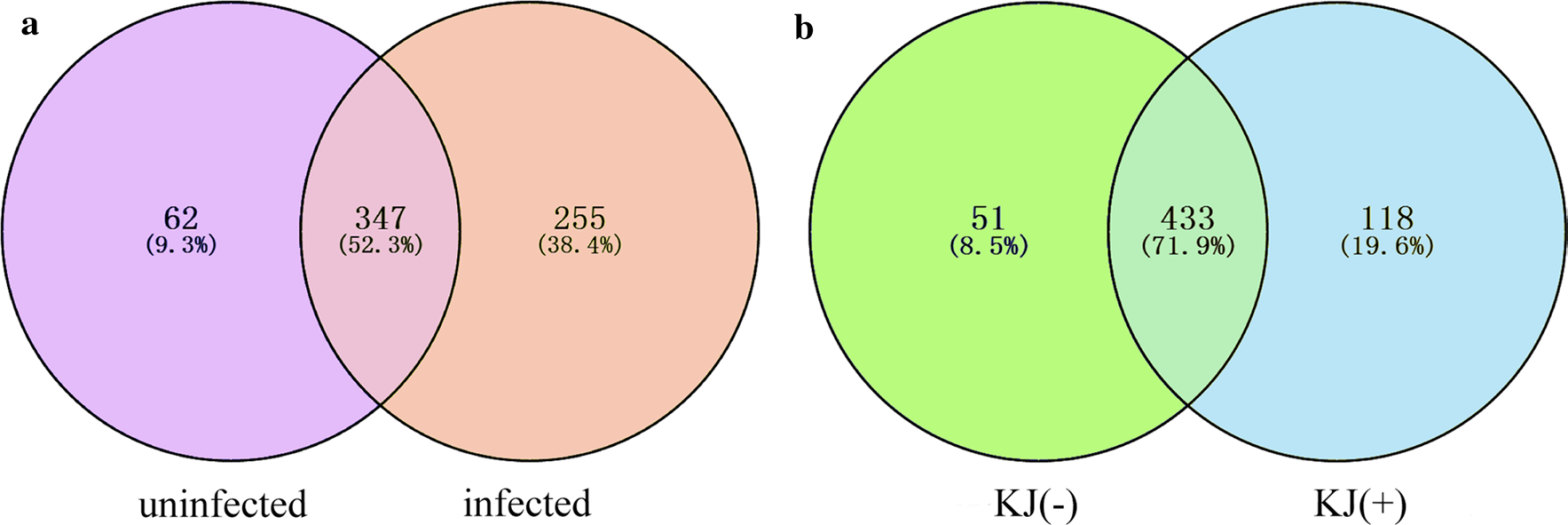
Numbers and sequence proportions of Shared OTUs between uninfected and infected groups (**a**), and KJ(−) and KJ(+) groups (**b**)

On the basis of a 97% similarity threshold, 664 OTUs were identified at the phylum level, and 49.6% (329) of OTUs were assigned to the genus level (Additional file 4: Table S2). At the phylum level, differences in the dominant microbiota between uninfected and infected groups were not significant: Proteobacteria (58.2 ± 35.3% *vs* 67.7 ± 29.1%; *t*_(23)_ = − 0.574, *P* = 0.572), Fusobacteria (19.2 ± 28.3% *vs* 19.2 ± 21.9%; *t*_(23)_ = -0.001, *P* = 0.999), Tenericutes (14.6 ± 23.5% *vs* 6.7 ± 18.9%; *t*_(23)_ = 0.733, *P* = 0.472) (Fig. 1a). At the genus/family level, *Aeromonadaceae*, *Cetobacterium*, *Enterobacteriaceae* and *Mycoplasma* were the relatively abundant taxa (higher than 1%) both in infected and uninfected common carp. There were no significant differences in the relative abundance of the four genera/families between uninfected and infected groups (Fig. 1b; *Aeromonadaceae*: *t*_(23)_ = − 0.075, *P* = 0.941; *Cetobacterium*: *t*_(23)_ = 0.245, *P* = 0.809; *Enterobacteriaceae*: *t*_(23)_ = − 0.463, *P* = 0.648; *Mycoplasma*: *t*_(23)_ = − 0.507, *P* = 0.617). Compared with the uninfected group, the relative abundance of five genera, *Salinivibrio*, *U114* (Fusobacteria), *Clostridium*, *Lawsonia* and *Bacteroides*, was higher in infected common carp (Fig. 1b). The Lefse analysis found that a total of 22 taxa displayed significant differences in their abundance between uninfected and infected common carp at a stringent cutoff value (absolute LDA score log_10_ ≥ 2.0) (Fig. 2a). Among the identified OTUs, 409 were shared by the uninfected group samples (61.6% of sequences), and 601 were shared by the infected group samples (90.5%). Among these, 346 OTUs were shared by the two groups (Fig. 3a).

### Alpha diversity of microbiota in infected and uninfected common carp

**Fig. 4:**
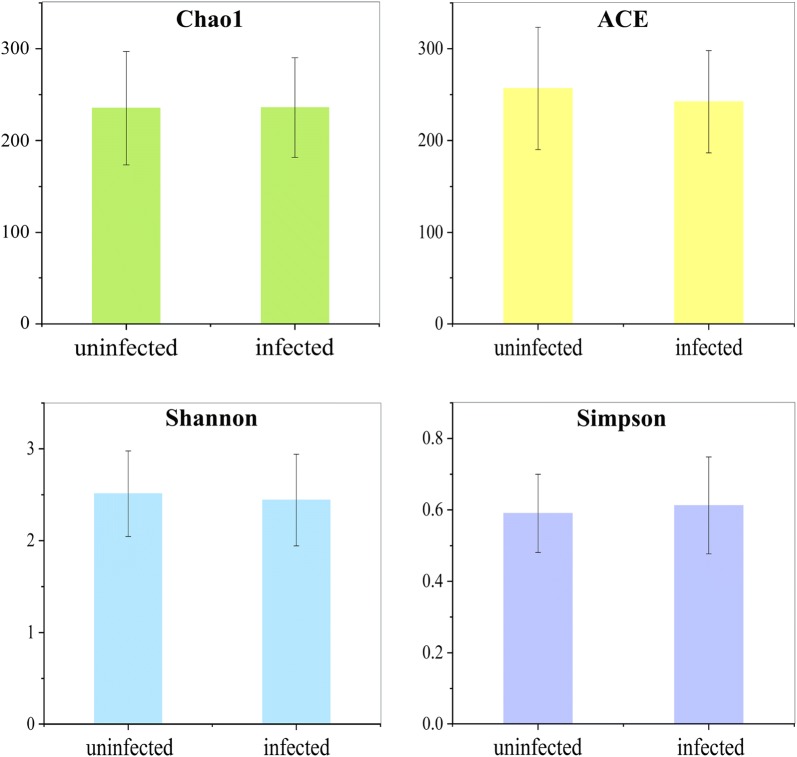
Alpha diversity of microbial communities in the intestines of uninfected and infected common carp

There were no significant differences in richness (Chao1 and ACE) and diversity (Shannon, Simpson) between the infected and uninfected groups (Chao1: *t*_(23)_ = − 0.021, *P* = 0.984; ACE: *t*_(23)_ = 0.447, *P* = 0.659; Shannon: *t*_(23)_ = 0.237, *P* = 0.815; Simpson: *t*_(23)_ = − 0.265, *P* = 0.793) (Fig. 4).

### Beta diversity of microbiota in infected and uninfected common carp

**Fig. 5:**
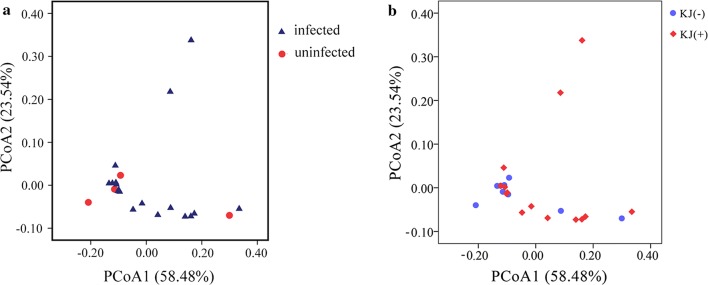
Principal coordinates analysis (PCoA) of bacterial community structures. **a** Triangles and dots represent the samples in infected and uninfected groups, respectively. **b** Dots and rhombuses represent the samples in KJ(−) and KJ(+) groups, respectively

PERMANOVA indicated that there was no significant difference in the composition of microbial communities between the infected and uninfected groups (*F*_(1,21)_ = 0.5262, *P* = 0.821). PCoA analysis also showed that bacterial communities of the two groups could not be clearly distinguished (Fig. 5a). However, the Mantel test showed that the abundance of *K. japonensis* had a significant effect on the bacterial composition (*P* = 0.015), while the abundance of *A. tenuicollis* had hardly any influence on the composition of gut microbiota (*P* = 0.954). Therefore, composition and diversity of microbiota were further analyzed between the groups infected KJ(+) and uninfected KJ(−) with *K. japonensis* (Table 1).

### Microbiota composition in KJ(+) and KJ(−) groups

Ratios of dominant microbial taxa at the phylum level differed between KJ(−) and KJ(+) groups, but not significantly: Proteobacteria (74.2 ± 28.6% *vs* 60.8 ± 30.2%; *t*_(23)_ = 1.059, *P* = 0.302), Fusobacteria (13.9 ± 20.4% *vs* 22.7 ± 23.7%; *t*_(23)_ = − 0.917, *P* = 0.370) and Tenericutes (6.6 ± 16.3% *vs* 9.0 ± 21.7%; *t*_(23)_ = − 0.284, *P* = 0.779) (Fig. 1c). Similarly, there were also no significant differences between KJ(−) and KJ(+) groups at the genus/family level (only relatively abundant taxa, higher than 1%, were tested): *Aeromonadaceae* (*t*_(23)_ = 0.651, *P* = 0.522), *Enterobacteriaceae* (*t*_(23)_ = − 0.480, *P* = 0.636), *Cetobacterium* (*t*_(23)_ = 0.519, *P* = 0.609), *U114* (*t*_(23)_ = − 0.747, *P* = 0.464) and *Salinivibrio* (*t*_(23)_ = 0.196, *P* = 0.864) (Fig. 1d). The relative abundance of the genera *Mycoplasma* (*t*_(23)_ = 1.356, *P* = 0.198), *Clostridium* (*t*_(23)_ = 2.228, *P* = 0.042), *Lawsonia* (*t*_(23)_ = 1.196, *P* = 0.252), *Bacteroides* (*t*_(23)_ = 0.723, *P* = 0.477) and *Plesiomonas* (*t*_(23)_ = 1.288, *P* = 0.212) was higher in the KJ(+) group than in KJ(−) group (Fig. 1d). The Lefse analysis indicated that a total of 21 taxa displayed a significant difference in their abundance between KJ(−) and KJ(+) groups at a stringent cutoff value (absolute LDA score log_10_ ≥ 2.0) (Fig. 2b). OTUs were determined to investigate shared microbial communities: 484 OTUs were shared by the samples from the KJ(−) group (80.4% of sequences), and 551 OTUs were shared by samples from the KJ(+) group (91.5%). Among these, 433 OTUs were shared between the two groups (Fig. 3b).

### Alpha diversity of microbiota in KJ(−) and KJ(+) groups

**Fig. 6:**
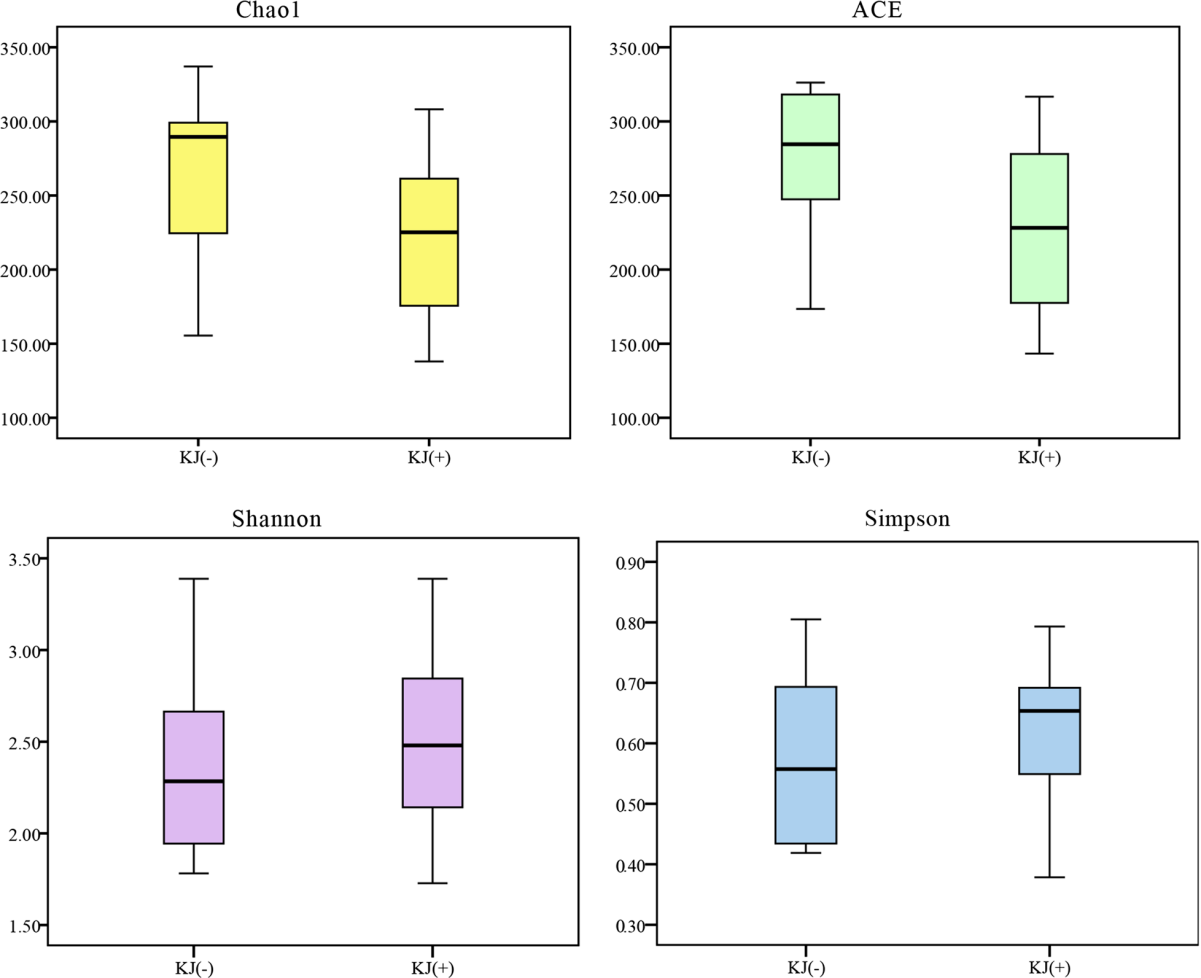
Alpha diversity of microbial communities in KJ(−) and KJ(+) groups

There were also no significant differences in richness (Chao1 and ACE) and diversity (Shannon, Simpson) between KJ(−) and KJ(+) groups (Chao1: *t*_(23)_ = 1.635, *P* = 0.117; ACE: *t*_(23)_ = 1.728, *P* = 0.099; Shannon: *t*_(23)_ = − 0.539, *P* = 0.596; Simpson: *t*_(23)_ = − 1.051, *P* = 0.305) (Fig. 6).

### Beta diversity of microbiota in KJ(−) and KJ(+) groups

PERMANOVA indicated that there was also no significant difference in the composition of microbial communities between KJ(−) and KJ(+) groups (*F*_(1,21)_ = 0.433, *P* = 0.883). PCoA analysis also showed that bacterial communities of KJ(−) and KJ(+) groups could not be clearly distinguished (Fig. 5b). Pearson correlation coefficient analysis indicated that the abundance of *K. japonensis* had an extremely significant (*P *< 0.001) positive correlation with the relative abundance of *Epulopiscium*, *U114*, *Bacteroides*, *Clostridium*, *Peptostreptococcaceae* (0.8< *r *< 0.9); and a significant correlation (*P *< 0.05) with *Enterobacteriaceae*, *Micrococcaceae*, *Rummeliibacillus*, *Lysinibacillus boronitolerans*, *Veillonellaceae*, *Oxalobacteraceae*, *Aeromonadaceae* (negative), *Marinibacillus* and *Chitinilyticum* (0.4< *r *< 0.7) (Table 2).

**Table 2:** Correlation coefficient analysis between the abundance of *Khawia japonensis* and abundance of each bacterium in the intestine of common carp (*Cyprinus carpio*)

Taxon	*P-*value	*r*
*Epulopiscium*	< 0.0001	0.87
*U114*	< 0.0001	0.86
*Clostridium*	< 0.0001	0.86
*Bacteroides*	< 0.0001	0.86
*Peptostreptococcaceae*	< 0.0001	0.84
*Enterobacteriaceae*	0.0003	0.68
*Micrococcaceae*	0.002	0.62
*Rummeliibacillus*	0.003	0.59
*Lysinibacillus boronitolerans*	0.003	0.59
*Veillonellaceae*	0.006	0.55
*Oxalobacteraceae*	0.015	0.50
*Aeromonadaceae*	0.019	− 0.49
*Marinibacillus*	0.022	0.48
*Chitinilyticum*	0.024	0.47

## Discussion

Proteobacteria, Fusobacteria and Tenericutes were the dominant microbiota in the hindgut of common carp in our study, which is in disagreement with a previous study of intestinal microbiota of common carp [41]. This may be attributable to differences in diet [42], as the common carp in the previous study was fed a commercial feed, whereas we used a diet composed of crustaceans, snails and detritus in our study.

Increased alpha diversity of the intestinal microbiota is generally associated with a “healthy” gut homeostasis [1]. Although several studies have reported a marked decrease in alpha diversity in animals infected (the acute phase of infection) by parasitic helminths [5–7], in most studies conducted to date in a range of animal-helminth systems, the alpha diversity of the gut microbiota remained unchanged following a parasitic infection [9, 11, 18, 21, 43, 44]. In line with this, we did not observe a significant change in alpha diversity in tapeworm-infected common carp in our study. This indicates that parasitic infections are likely to affect only a small fraction of the intestinal microbiota [9, 11]. In addition, it may also be related to the small number of uninfected samples in our trial.

Although two tapeworm species (parasitising the common carp) were discovered in our study, only the abundance of *K. japonensis* had a significant effect on the composition of microbiota, while the abundance of *A. tenuicollis* had almost no influence on the composition of microbiota. The relative body surface area of *K. japonensis* is about two to three times larger than that of *A. tenuicollis* [23, 45], so the former species can host a much larger number of bacteria. In addition, type 2 immune responses induced by helminth infections can alter the host’s metabolic functions, as well as modify bacterial microbiota populations, but the magnitude of these changes varies among helminth species [3, 21]. We hypothesise that these two factors may explain the much more pronounced effect of *K. japonensis* on the composition of gut microbiota (compared to *A. tenuicollis*).

Although none of the observed differences in the composition of intestinal microbiota between infected and uninfected common carp were statistically significant, it may be worth noting that tapeworm infection was associated with an increased relative abundance of two pathogenic bacterial taxa: *Lawsonia* and *Plesiomonas*. *Lawsonia intracellularis* (family *Desulfovibrionaceae*), the only member of the genus, is an obligate intracellular parasite of intestinal cells, which causes proliferative enteropathy in pigs and some other mammals [46, 47]. *Plesiomonas shigelloides*, also the sole member of the genus, is ubiquitous in surface water and soil, and can cause gastroenteritis and extraintestinal infections [48, 49]. Thus the increased relative abundance of *Lawsonia* and *Plesiomonas* may contribute to infection by the caryophyllidean tapeworms.

*Khawia japonensis* had a significant positive correlation with *U114*, *Epulopiscium*, *Bacteroides*, *Clostridium*, *Peptostreptococcaceae*, *Rummeliibacillus*, *Lysinibacillus boronitolerans*, *Marinibacillus* and *Chitinilyticum* in our study. *U114* might have a role in the regulation of bile acids metabolism [50]; *Epulopiscium* may be able to break down carbohydrates and complex hemicellulose [51]; *Bacteroides* has a capacity of breaking down polysaccharides [52]; *Clostridium* ferments polysaccharides and proteins to produce alcohols and short-chain fatty acids [53]; *Peptostreptococcaceae* can utilize proteinaceous substrates and carbohydrates [54]; *Rummeliibacillus* can hydrolyze gelatin and produce acids [55]; *Lysinibacillus* *boronitolerans* produces nitrilase, converses iminodiacetonitrile (IDAN) to iminodiacetic acid (IDA) [56], and degrades ioxynil octanoate herbicide [57]; *Marinibacillus* utilizes cellobiose, trehalose and xylose [58]; and finally, *Chitinilyticum* degrades chitin [59]. All these microbial taxa are very important for the digestion of polysaccharides and proteins. In addition, due to the lack of guts, cestodes absorb nutrients through the tegument [60]. There is a large amount of microtriche on the surface of teguments of cestodes, and bacteria are abundant around the microtriche [61–64]. These bacteria can produce enzymes that hydrolyze carbohydrates, disaccharides and proteins [65, 66]. Therefore, the microbiota related to the tapeworm infection is likely to help the cestodes absorb nutrients through the tegument. Whether the microbiota is associated strictly with the tegument of cestodes needs to be further investigated.

Some opportunistic pathogens, such as *Enterobacteriaceae*, *Micrococcaceae*, *Veillonellaceae*, *Oxalobacteraceae* and *Aeromonadaceae*, were also significantly correlated with the infection with *K. japonensis*. Cestodes embed themselves into the intestinal wall via their scolex, leading to blistering, bleeding, and inflammation of the intestinal mucosa, which can be followed by secondary infections by other pathogens [30]. However, previous studies have shown, and our results corroborated, that common carp infected by *K. japonensis* does not exhibit observable pathological changes [24]. Species of Clostridia (*Clostridium* especially) are known to tighten the epithelial barrier and decrease the propensity for allergy [67, 68]. The relative abundance of *Clostridium* was strikingly increased in our study. Therefore, we hypothesize that the increased abundance of *Clostridium* probably reduced the damage to the intestinal epithelium caused by the tapeworms, which may also be the reason why no obvious pathological changes were observed. However, the ability of *Clostridium* to reduce allergic reactions in the gut of common carp would first have to be proven by further experiments.

## Conclusions

Tapeworm colonization in common carp did not affect the microbial diversity, but it altered the microbial composition at the genus level. Compared with *A. tenuicollis*, *K. japonensis* had a greater impact on the composition of intestinal microbiota. Some of the microbial taxa associated with *K. japonensis* may contribute to nutrient digestion and absorption by the tapeworm. *Khawia japonensis* infection was associated with increased ratios of some pathogenic bacteria (*Lawsonia* and *Plesiomonas*), but it also increased the ratio of *Clostridium*, which may have reduced the allergic reaction and pathological changes caused by pathogenic bacteria.

## Additional files

**Additional file 1: Figure S1.** Morphological characteristics of *Atractolytocestus tenuicollis* in the intestine of common carp (*Cyprinus carpio*) from China.

**Additional file 2: Figure S2.** Morphological characteristics of *Khawia japonensis* in the intestine of common carp (*Cyprinus carpio*) from China.

**Additional file 3: Table S1.** Maturity of the two tapeworms *Khawia japonensis* and *Atractolytocestus tenuicollis* in the intestine of common carp (*Cyprinus carpio*) from China.

**Additional file 4: Table S2.** Complete OTU table of gut microbiota at the genus level in the intestine of common carp (*Cyprinus carpio*).

## Data Availability

The data supporting the findings of this study are included within the article. The sequences were deposited in the NCBI SRA database under the accession number SRP158810.

## Abbreviations

GI: gastrointestinal
OUT: operational taxonomic unit
Lefse: linear discriminant analysis coupled with effect size
PCoA: principal coordinates analysis plots
PERMANOVA: permutational multivariate analyses of variance
IDAN: iminodiacetonitrile
IDA: iminodiacetic acid
