# Supplementary figures and images for: Effect of intestinal tapeworms on the gut microbiota of the common carp, Cyprinus carpio
Source: Parasit Vectors. 2019 May 22;12:252. doi: 10.1186/s13071-019-3510-z (PMC6530175; doi:10.1186/s13071-019-3510-z)

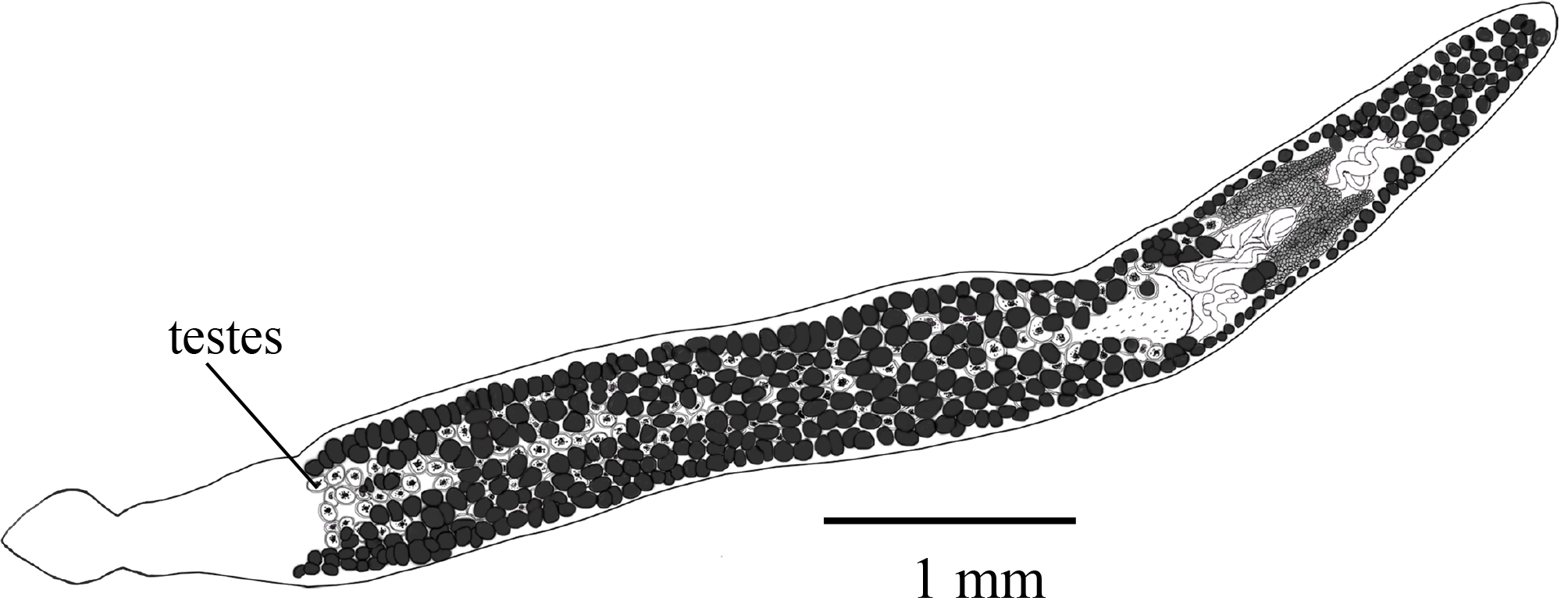

Supplement: Supplementary file 1 — Additional file 1: Figure S1. Morphological characteristics of Atractolytocestus tenuicollis in the intestine of common carp (Cyprinus carpio) from China. [file 13071_2019_3510_MOESM1_ESM.tif]

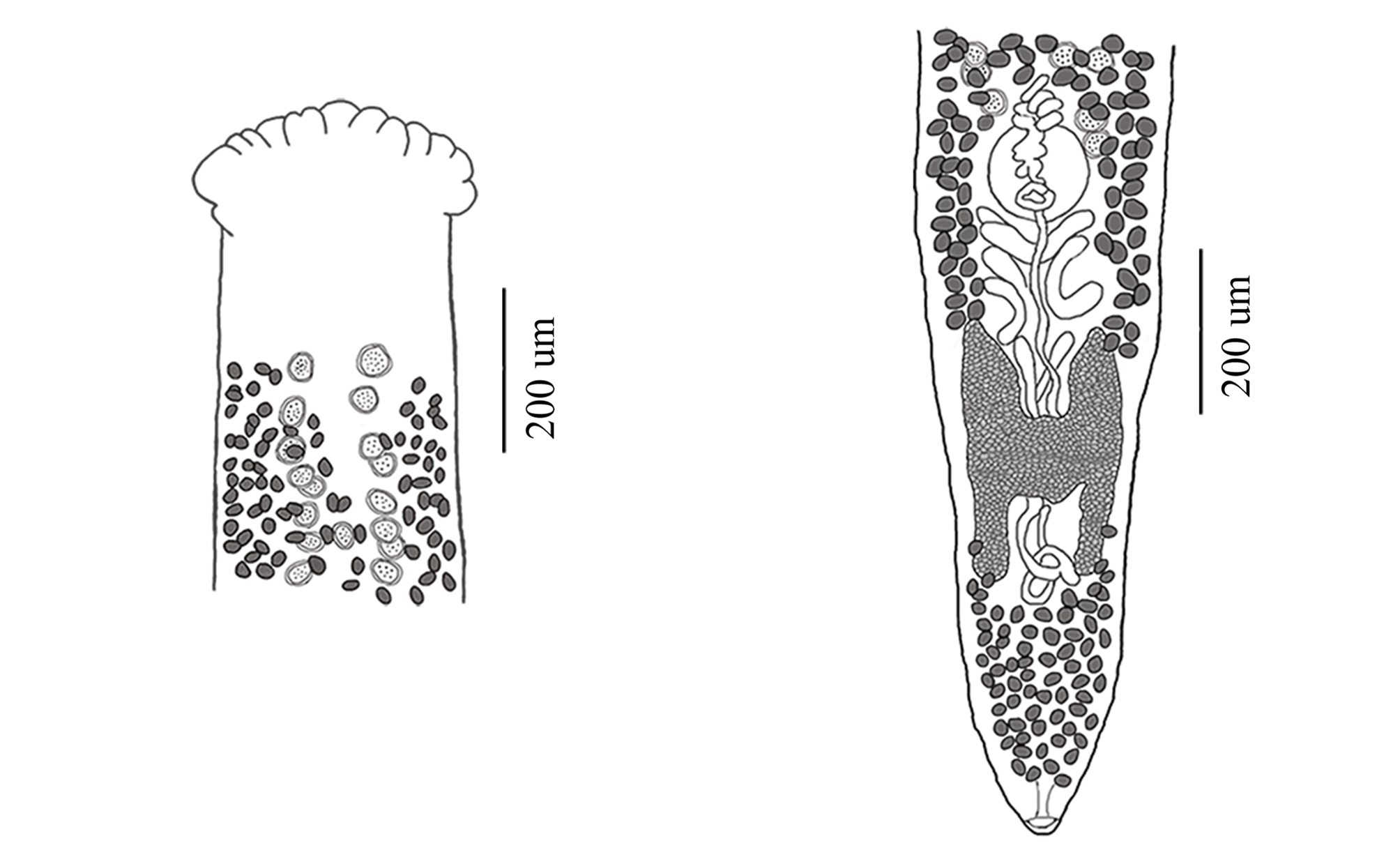

Supplement: Supplementary file 2 — Additional file 2: Figure S2. Morphological characteristics of Khawia japonensis in the intestine of common carp (Cyprinus carpio) from China. [file 13071_2019_3510_MOESM2_ESM.tif]
